# Supplementary material for: Reviewers’ Ratings and Bibliometric Indicators: Hand in Hand When Assessing Over Research Proposals?
Source: PLoS One. 2013 Jun 28;8(6):e68258. doi: 10.1371/journal.pone.0068258 (PMC3695904; doi:10.1371/journal.pone.0068258)
Supplement: Materials S1 — (PDF) [file pone.0068258.s001.pdf]

S1. SUPPORTING MATERIALS TO MANUSCRIPT:

Reviewers' ratings and bibliometric indicators: hand in hand when assessing over research proposals?

Álvaro Cabezas-Clavijo[a], Nicolás Robinson-García[a], Manuel Escabias[b], Evaristo Jiménez-Contreras[a]

[a]*EC3: Evaluación de la Ciencia y la Comunicación Científica, Universidad de Granada, Granada, Spain. Email address: acabezasclavijo@gmail.com; {elrobin, evaristo}@ugr.es*

[b]*Departamento de Estadística e Investigación Operativa, Universidad de Granada, Granada, Spain. Email address: escabias@ugr.es*

1. Supporting Materials to subsection *'Description of referees' ratings, bibliometric indicators and granted vs. rejected distribution of grant proposals'* in the '*Results*' section.

Figures S1-S11 show box plots of the distribution of granted vs. rejected proposals according to bibliometric indicators and referees' ratings. Boxes in red show the distribution of granted proposals. Boxes in blue show the distribution of rejected proposals

Figure S1. Distribution of the research output of PIs for granted and rejected proposals

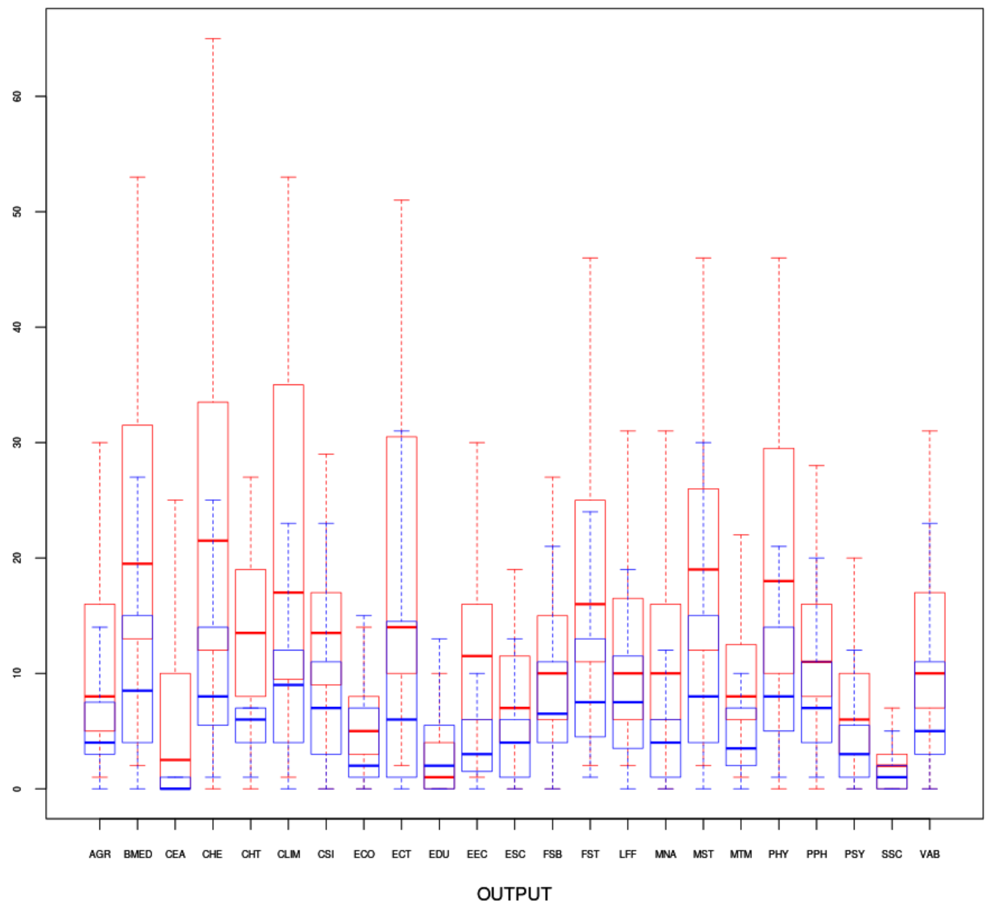

Figure S2. Distribution of the citation average of PIs for granted and rejected proposals

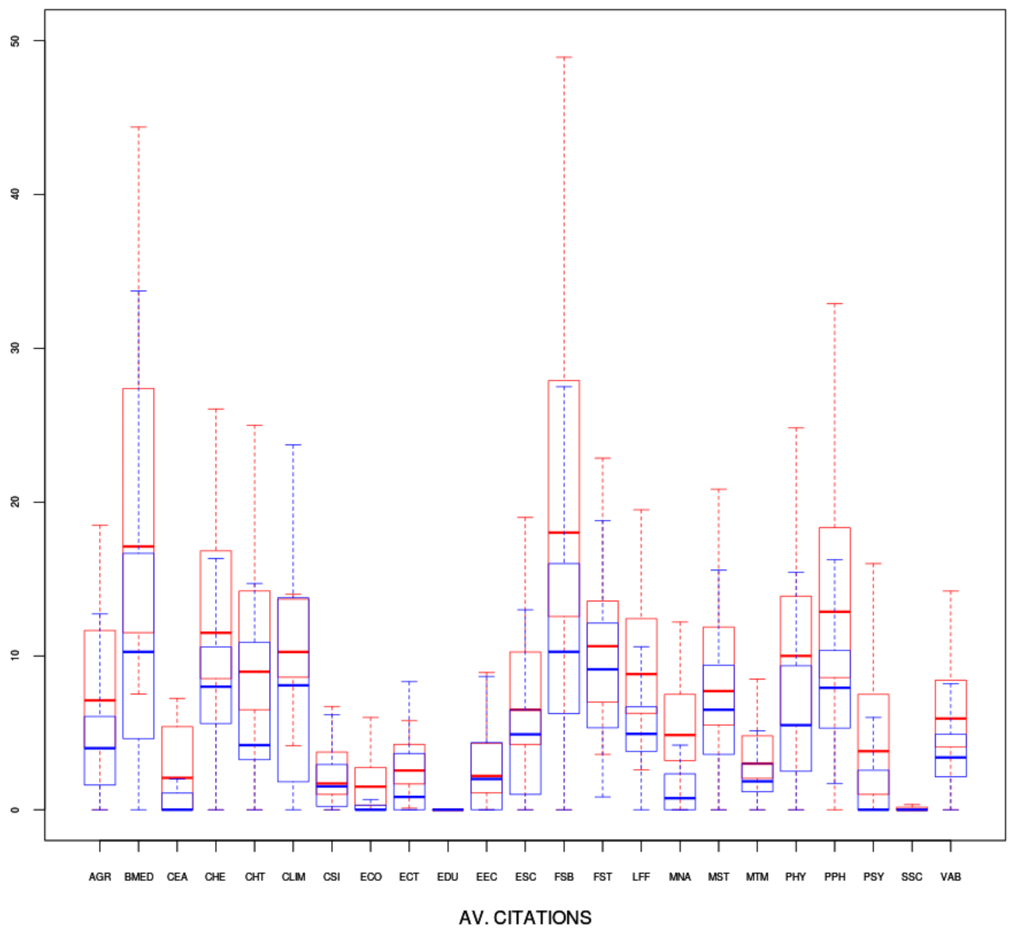

Figure S3. Distribution of the number of citations received by PIs for granted and rejected proposals

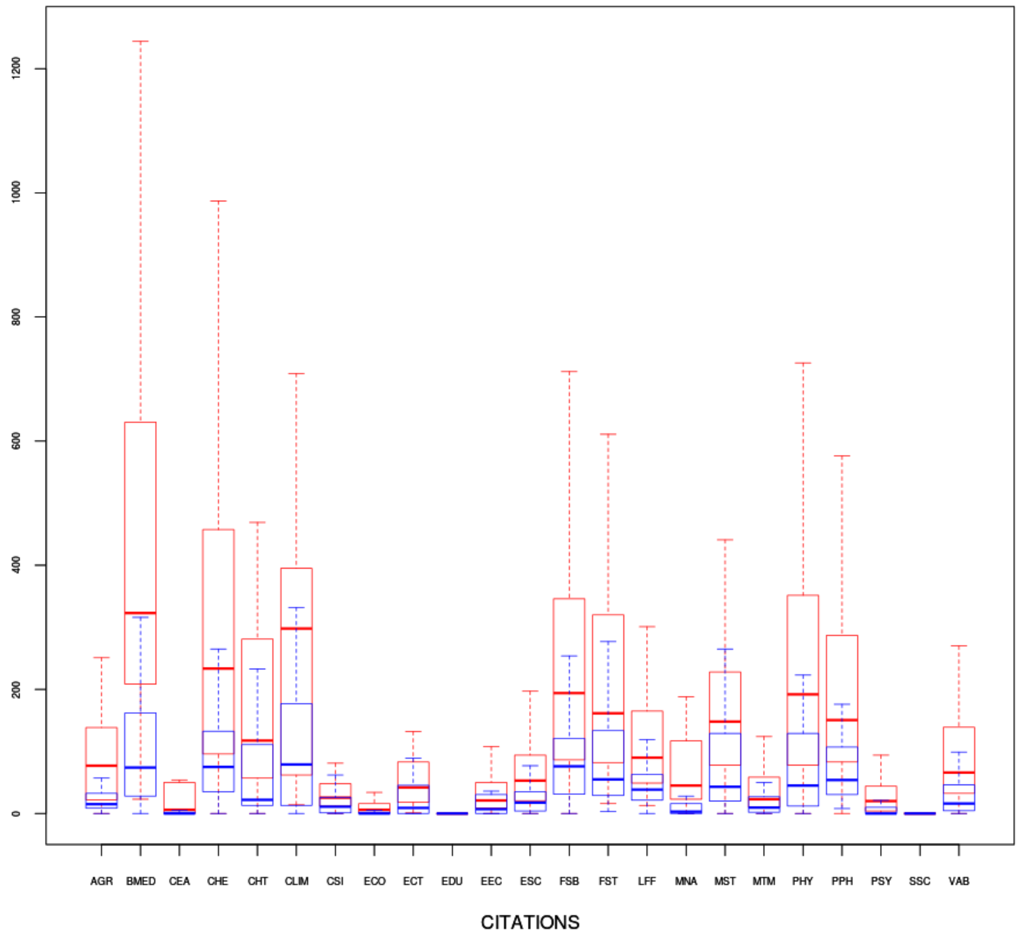

Figure S4. Distribution of the % of papers published in Q1 journals by PIs for granted and rejected proposals

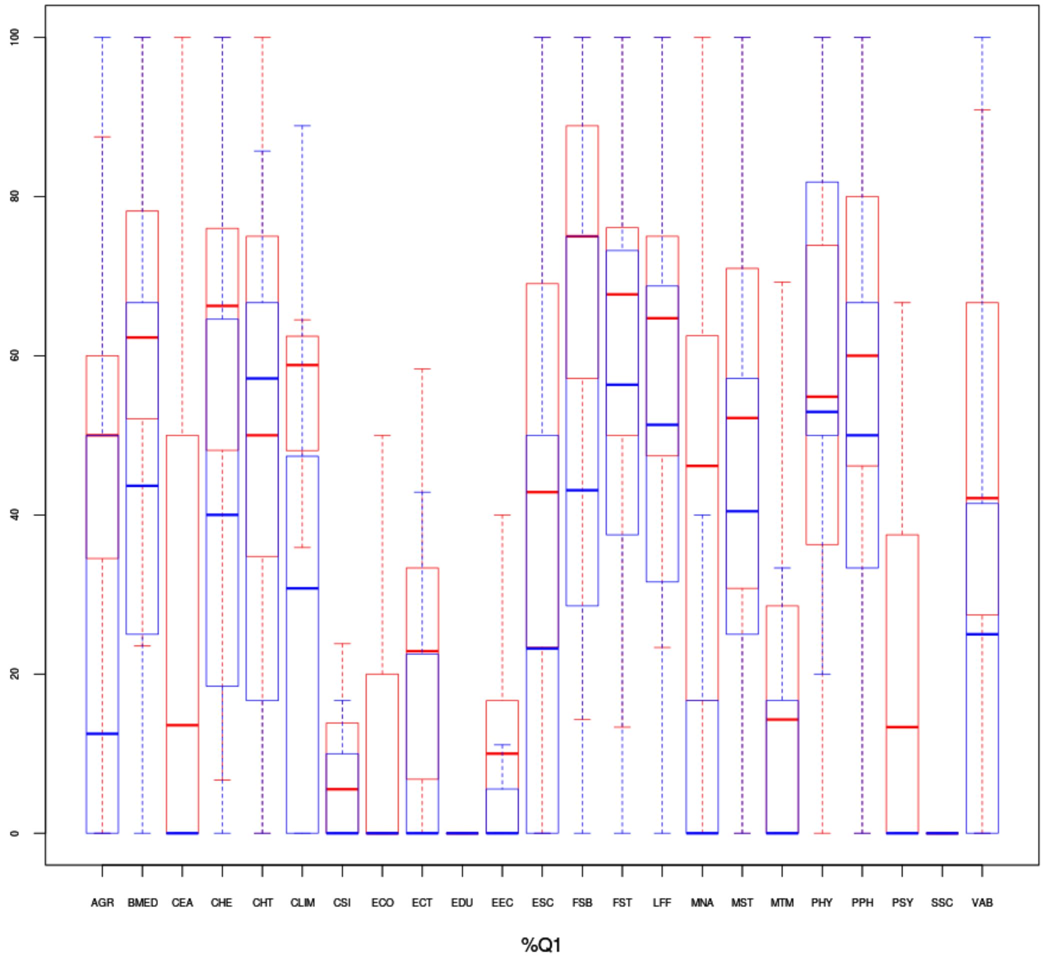

Figure S5. Distribution of the number of Q1 papers published by PIs for granted and rejected proposals

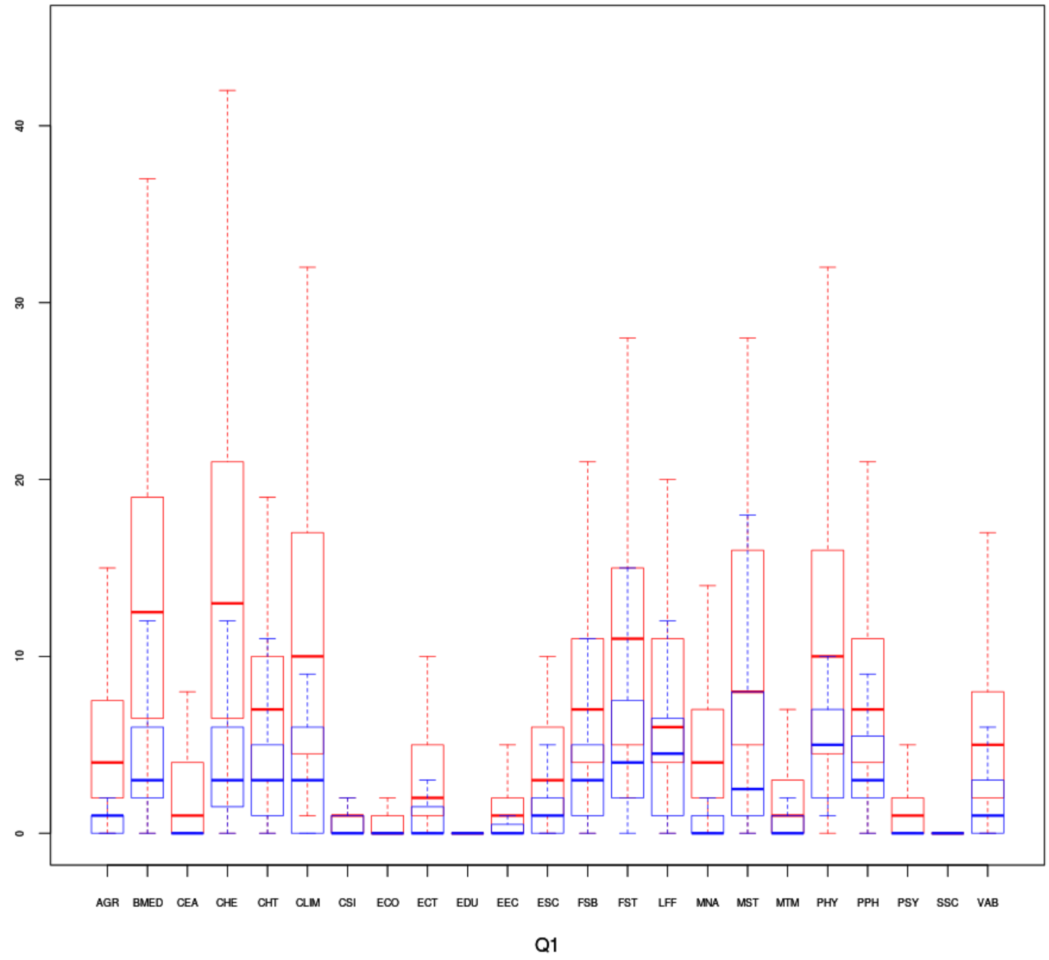

Figure S6. Distribution of the total ratings for granted and rejected proposals

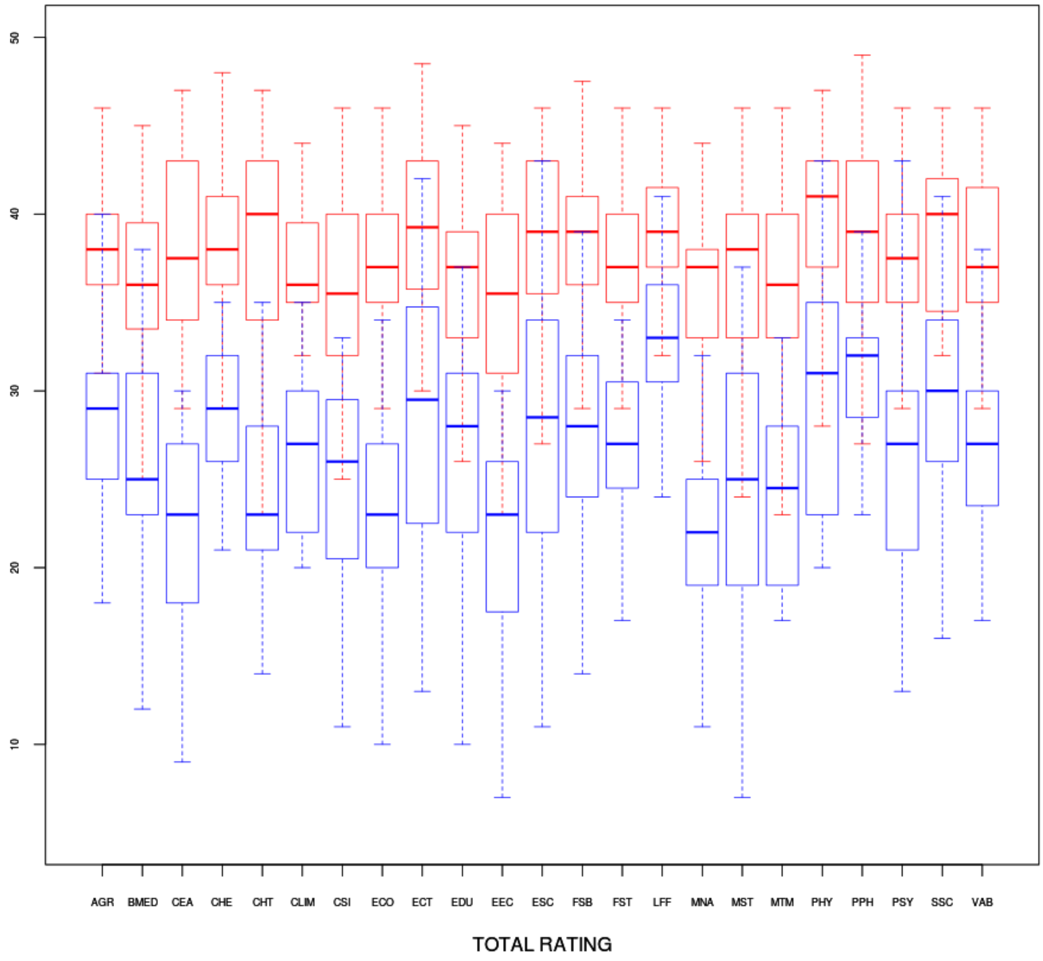

Figure S7. Distribution of the PIs' CV rating for granted and rejected proposals

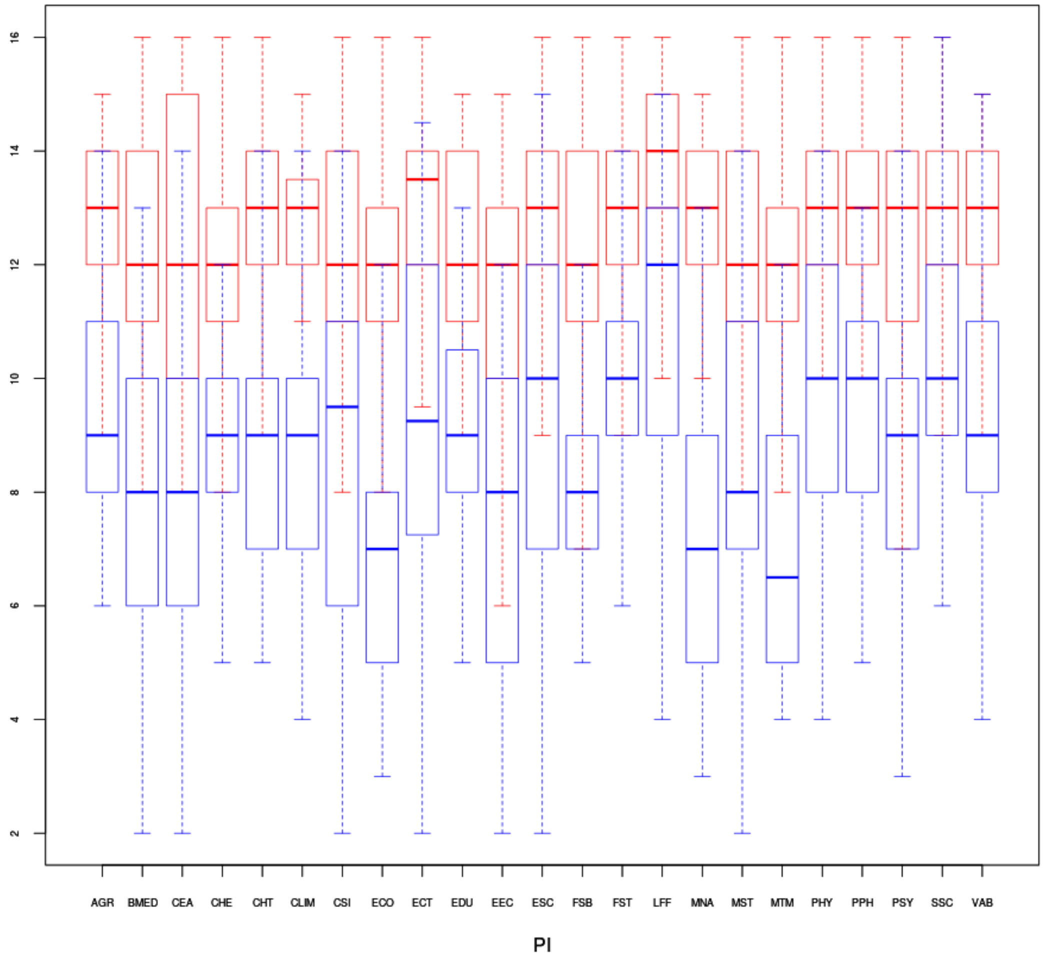

Figure S8. Distribution of the research teams' ratings for granted and rejected proposals

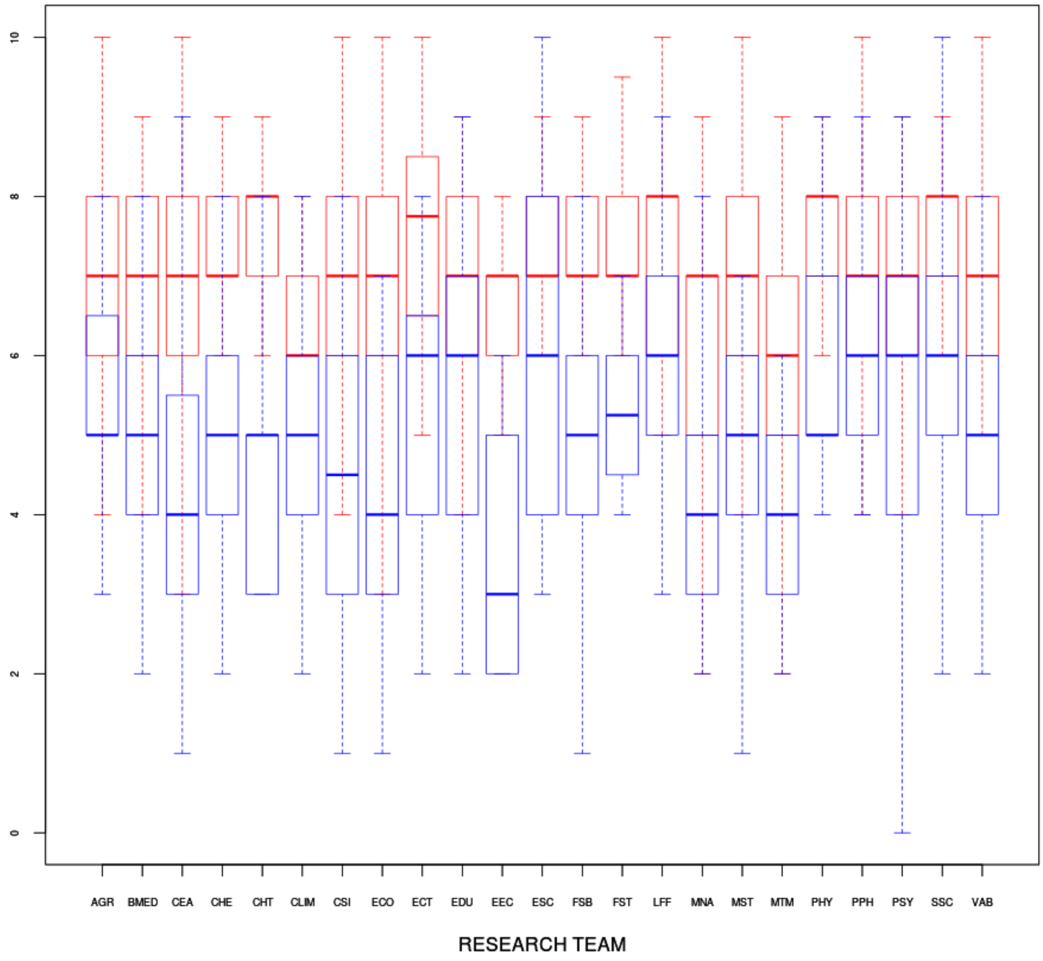

Figure S9. Distribution of the goals' ratings for granted and rejected proposals

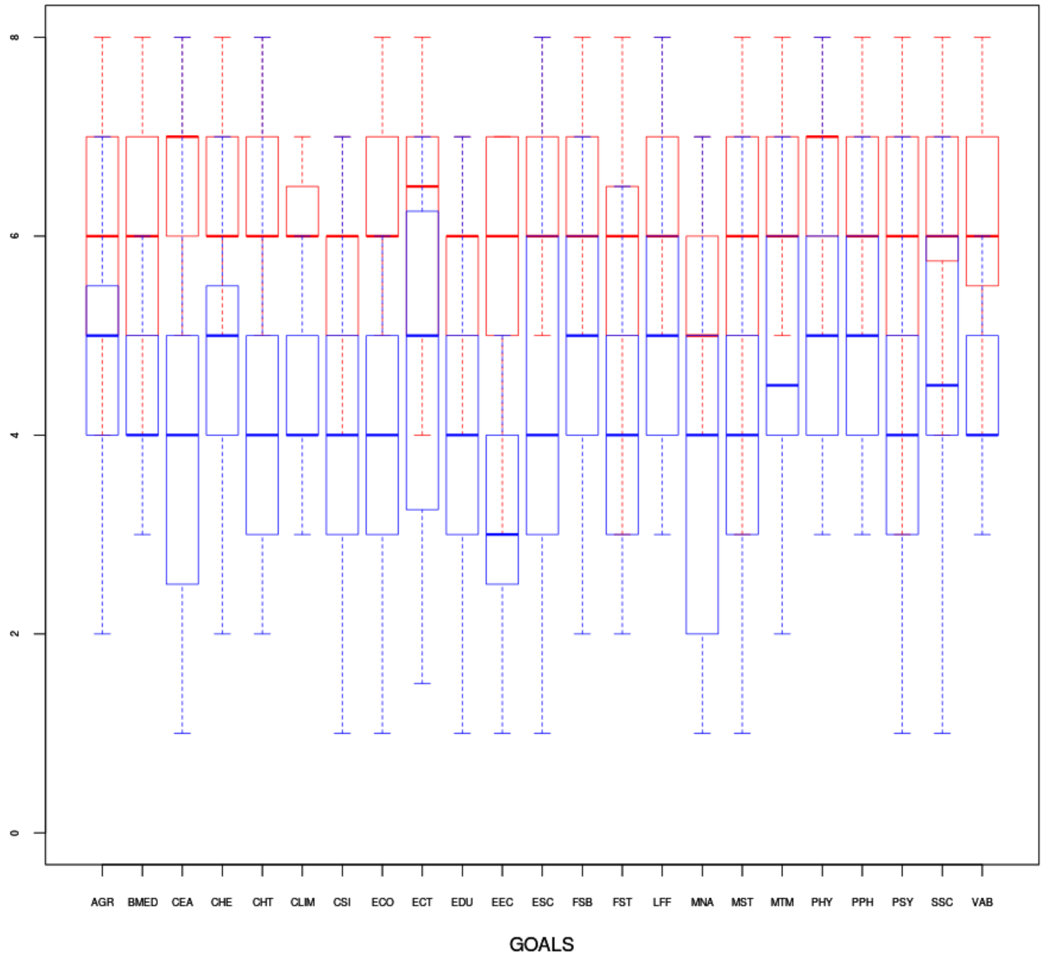

Figure S10. Distribution of the relevance's ratings for granted and rejected proposals

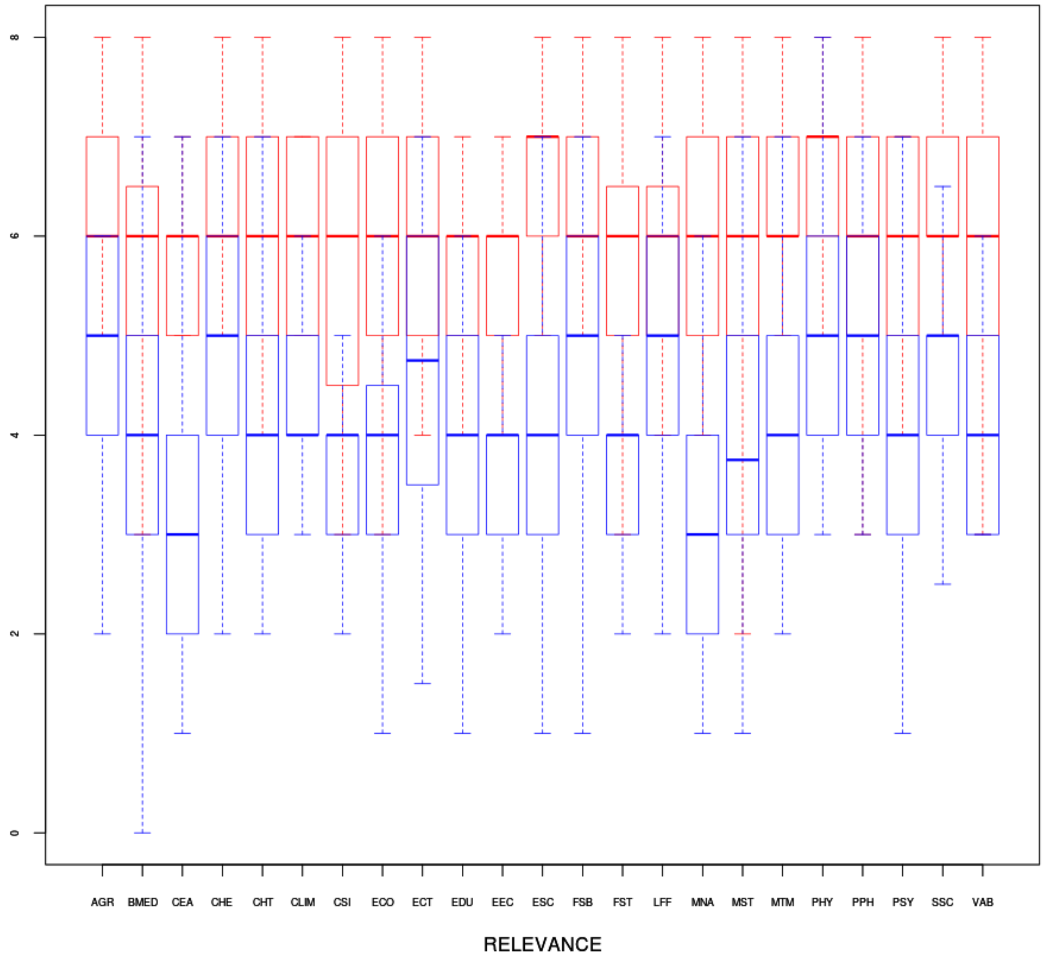

Figure S11. Distribution of the viability's ratings for granted and rejected proposals

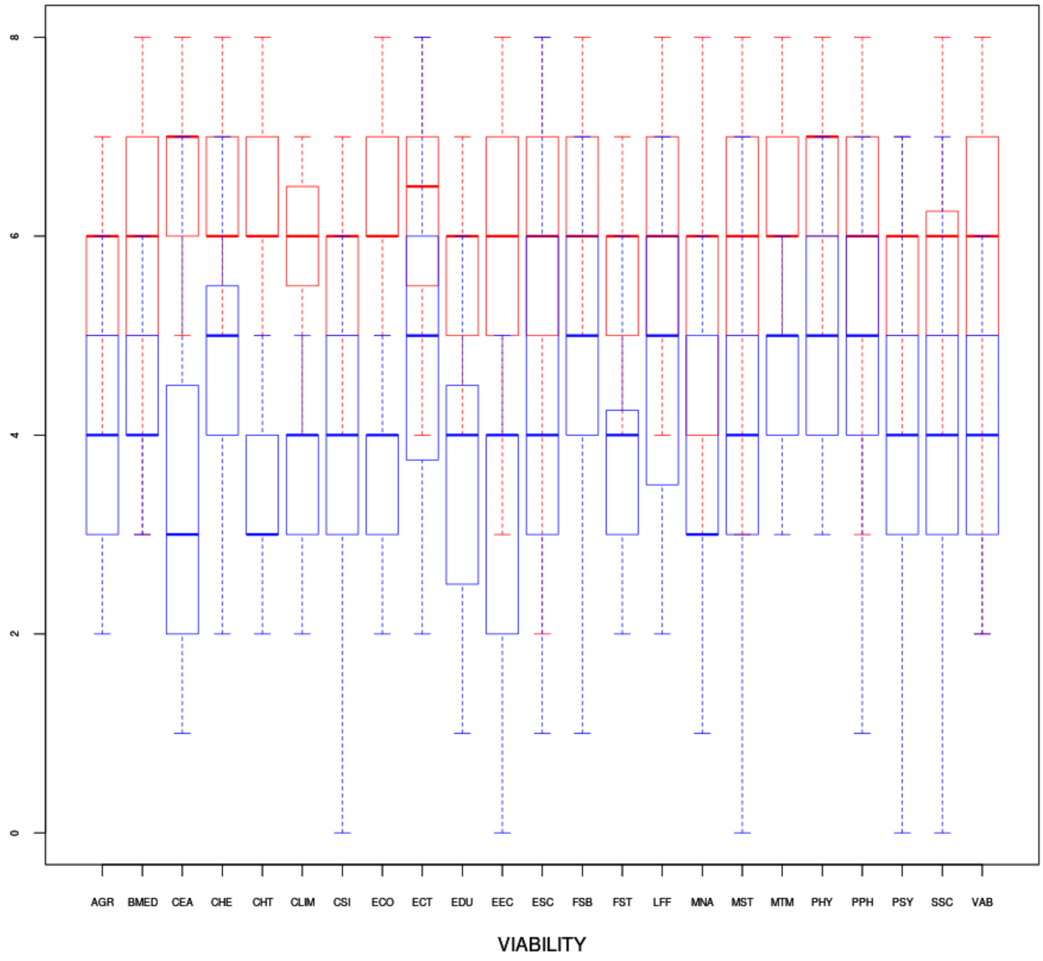

2. Supporting Materials to Table 3 in section *'Description of referees' ratings, bibliometric indicators and granted vs. rejected distribution of grant proposals'*

Table S1. Fits of the linear regression analysis to predict the concession of research proposals according to ratings to each of the sections reviewed by referees. Variables selected by the stepwise method. The area under the ROC curve, the Correct Classification Rate and the coefficient of determination (R2) are included

Area	Selected Variables	ROC	R2	CCR
**AGR**	I=2.47E-06, Viability=3.50, PI=1.82	0.93	0.68	0.88
**BMED**	I=6.81E-08, PI=2.43 Viability=2.13 Goals=1.80	0.95	0.73	0.85
**CEA**	I=2.24E-04, Viability=5.02	0.94	0.75	0.92
**CHE**	I=5.48E-09, PI=2.13, Research team=2.78, Goals=2.72	0.96	0.75	0.89
**CHT**	I=1.30E-04, Viability=10.85, Goals=0.18, Relevance=4.41	0.96	0.76	0.91
**CLIM**	*	*	*	*
**CSI**	I=5.24E-08, PI=2.10, Goals=3.54, Research team=1.68	0.95	0.72	0.88
**ECO**	I=5.81E-10, PI=3.03, Goals=3.68 Viability=2.11	0.98	0.86	0.93
**ECT**	I=1.95E-05, PI=1.49, Research team=1.99	0.87	0.55	0.79
**EDU**	I=1.28E-05, Relevance=1.85, PI=1.63, Viability=1.76	0.91	0.61	0.81
**EEC**	I=3.68E-09, Viability=11.020, Research team=2.2317, PI=1.7350	0.98	0.84	0.93
**ESC**	I=7.07E-05, Relevance=1.92, PI=1.45, Research team=1.47	0.89	0.56	0.80
**FSB**	I=1.38E-07, PI=2.25, Goals=1.85, Research team=1.57, Viability=1.46	0.95	0.71	0.88
**FST**	I=2.80E-09, PI=2.10, Goals=3.25, Research team=2.52	0.96	0.77	0.86
**LFF**	I=5.47E-08, PI=2.03., Viability=2.10, Research team=1.82	0.88	0.56	0.78
**MNA**	I=2.82E-03, Relevance=2.13, PI=1.34	0.93	0.63	0.84
**MST**	I=1.21E-05, Research team=3.86, PI=1.49	0.93	0.65	0.86
**MTM**	I=1.56E-07, PI=1.93, Research team=1.71, Goals=4.40	0.96	0.73	0.90
**PHY**	I=3.56E-03, PI=1.39, Research team=1.67	0.87	0.41	0.83
**PPH**	I=2.01E-06, PI=2.38, Relevance=2.11	0.93	0.61	0.84
**PSY**	I=1.13E-06, PI=1.98, Viability=3.50	0.95	0.73	0.89
**SSC**	I=1.59E-06, Relevance=2.65, PI=1.55, Research team=1.50	0.91	0.60	0.83
**VAB**	I=2.65E-08, Relevance=1.79, PI=1.87, Research team=2.47, Viability=1.61	0.94	0.72	0.90

* The logistic model does not apply to the data

3. Supporting Materials to Table 6 in section *'Influence of bibliometric indicators on peers' ratings'*

Table S2. Fits of the linear regression analysis to predict the PIs' ratings according to the bibliometric indicators. Variables selected by the stepwise method. The coefficient of determination (R2) is included

Area	Selected variables	R2
**AGR**	I=10.28, Q1=0.17, %Q1=0.01	0.2300119
**BMED**	I=7.46, Q1=0.10, %Q1=0.03	0.2521384
**CEA**	I=9.03, OUTPUT=0.25	0.2071898
**CHE**	I=8.19, Q1=0.07, AV CITATIONS=0.13, CITATIONS=-0.003, OUTPUT=0.06	0.4214612
**CHT**	I=9.64, OUTPUT=0.15, %Q1=0.02, CITATIONS=-0.003	0.2668563
**CLIM**	I=7.12, Q1=0.18, %Q1=0.04	0.5279744
**CSI**	I=9.15, OUTPUT=0.12	0.1983169
**ECO**	I=7.81, CITATIONS=0.10, %Q1=0.03, OUTPUT=0.09	0.2832918
**ECT**	I=9.63, OUTPUT=0.12, %Q1=0.05, AV CITATIONS=-0.20	0.3169063
**EDU**	I=10.20	0.0000000
**EEC**	I=7.32, OUTPUT=0.23, %Q1=0.06	0.5119366
**EST**	I=9.43, OUTPUT=0.27, AV CITATIONS=0.17, CITATIONS=-0.01	0.2395585
**FSB**	I=9.99, Q1=0.39, OUTPUT=-0.13	0.2534194
**FST**	I=6.58, OUTPUT=0.27, %Q1=0.05, Q1=-0.29, CITATIONS=0.003	0.4561903
**LFF**	I=11.22, CITATIONS=0.01	0.3038341
**MNA**	I=7.29, OUTPUT=0.21, %Q1=0.05	0.5387693
**MST**	I=7.29, OUTPUT=0.17, %Q1=0.05, Q1=-0.16	0.3603444
**MTM**	I=8.21, OUTPUT=0.17, Q1=0.45	0.3690643
**PHY**	I=9.81, AV CITATIONS=0.16, OUTPUT=0.09, CITATIONS=-0.004	0.2761185
**PPH**	I=9.84, Q1=0.18, AV CITATIONS=0.06	0.2769550
**PSY**	I=8.41, AV CITATIONS=0.37, OUTPUT=0.20, %Q1=0.02, CITATIONS=-0.01	0.3735310
**SSC**	I=11.37, AV CITATIONS=0.52	0.0728664
**VAB**	I=9.54, CITATIONS=0.01, %Q1=0.03, OUTPUT=0.10, Q1=-0.16	0.2675128
